# Supplementary material for: Controlled irrigation suppresses methane emissions by reshaping the rhizosphere microbiomes in rice
Source: Microbiol Spectr. 2025 Dec 23;14(2):e02970-25. doi: 10.1128/spectrum.02970-25 (PMC12889033; doi:10.1128/spectrum.02970-25)
Supplement: Supplemental material — Figures S1 to S8; Tables S1 to S6. [file spectrum.02970-25-s0001.docx]

**Supplementary Material (Lau et al):**

**Supplementary Figures (S1-S8) and Supplementary Tables (S1-S6)**

**SUPPLEMENTARY FIGURES**

**
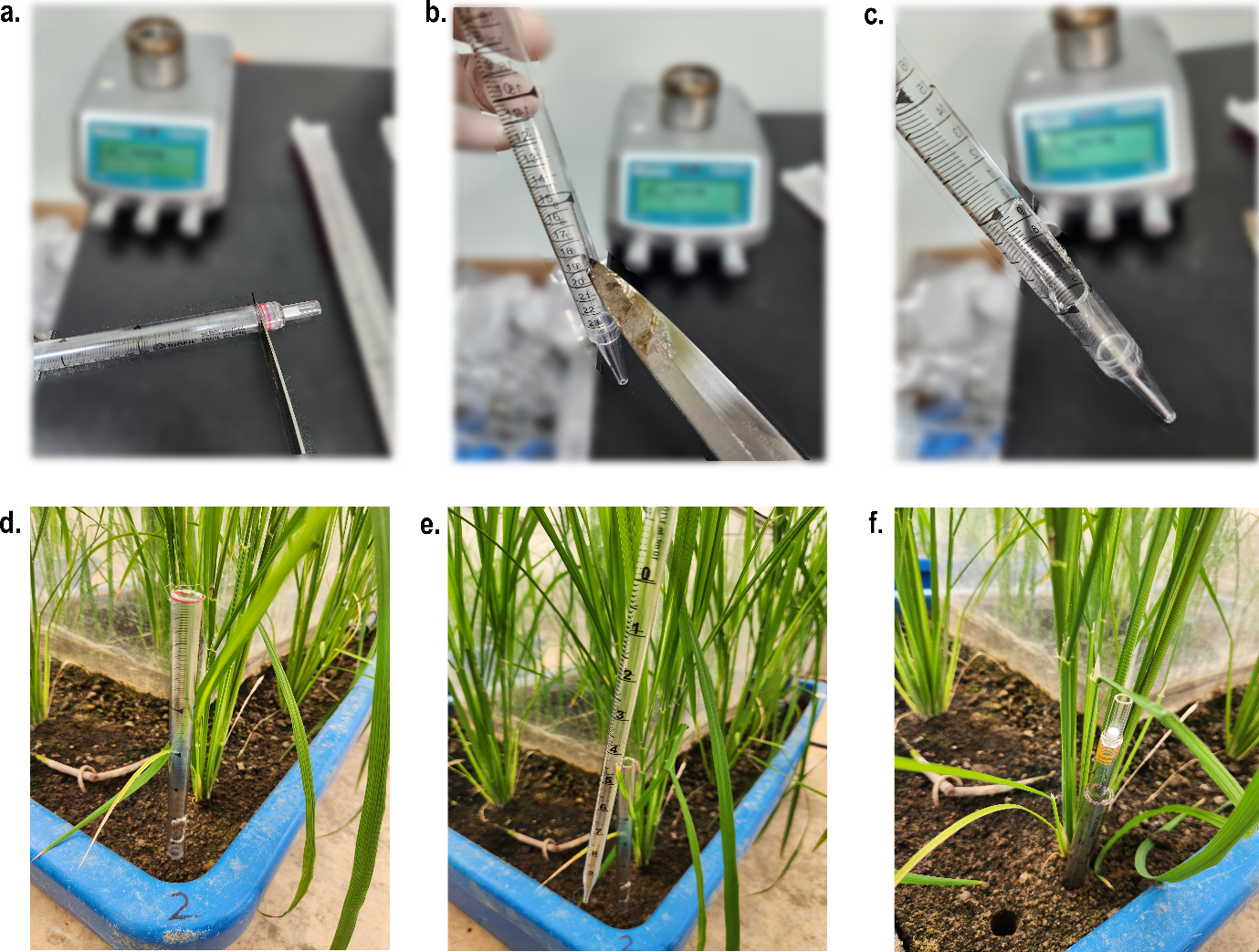
**

**Figure S1. Self-constructed soil probe used for rhizosphere sampling.** **a.** Heat-sterilized knife to cut the top of the 25 mL pipette, **b.** Creating a side opening around the 16- and 20-mL mark, **c.** the 25 mL pipette has the side opening similar to the commercial steel soil probes and **d.** the 25 mL body, **e.** a new 10 mL pipette was then inserted into the top of the 25mL body. **f.** The two-part soil probe was plunged into the soil next to the rice plant in close proximity to the stem. The 10 mL pipette was slowly removed once the probe reached the 15 cm depth.


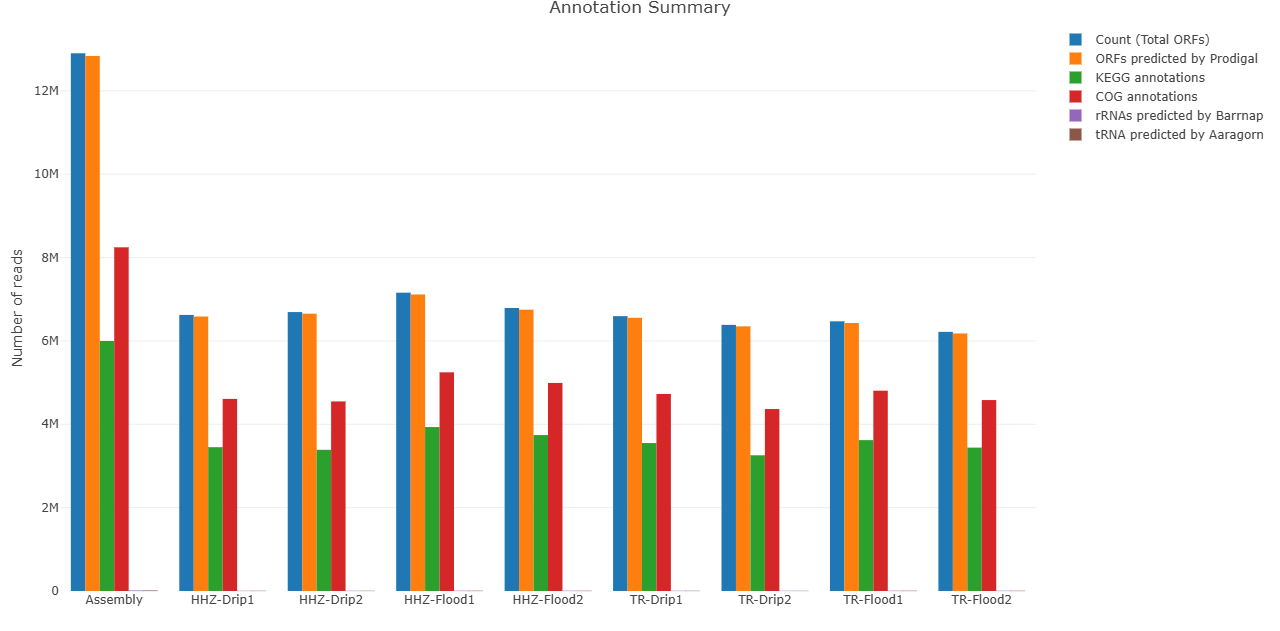


**Figure S2. Summary statistics of assembled metagenome contigs.** The bar chart presents a comparison of the number of reads for different genomic annotation results across an assembly and eight other samples grouped by irrigation conditions and replicates (HHZ-Drip, HHZ-Flood, TR-Drip, TR-Flood). The number of annotations for the assembled contigs is approximately double that of the raw input data from the other samples.

**
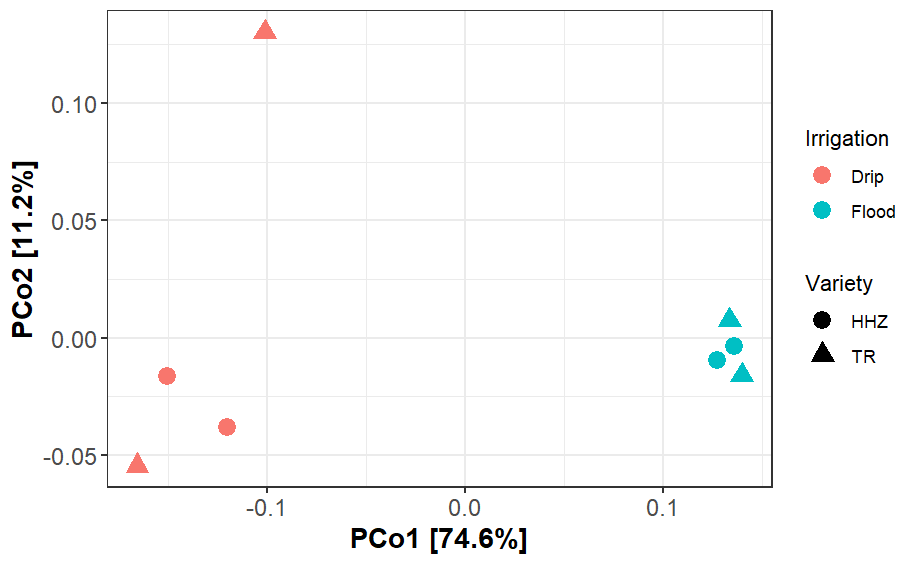
**

**Figure S3. Principal Coordinate Axis plot of drip and flood-irrigated soil microbiomes.** Flood-irrigated soil microbiomes are distinctly separated from the drip-irrigated rhizosphere microbiomes along the first principal coordinate axis, which accounts for 74.6% of the total variance. Orange represents drip-irrigation and cyan represents flood irrigation. Circles represent HHZ and triangles represent the TR rice variety, respectively.

**
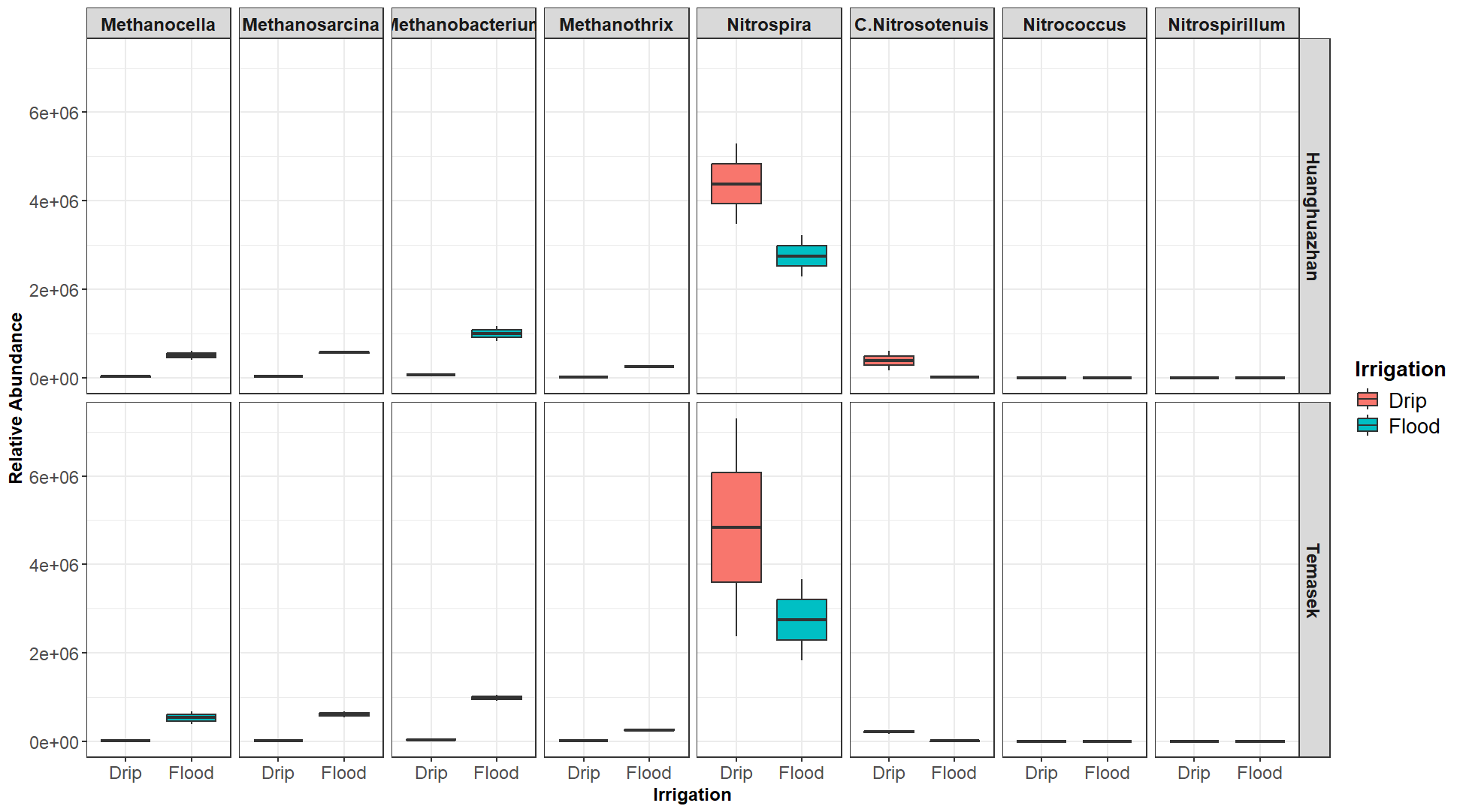

Figure S4. Differential taxa detected by MaAsLin2 that were enriched in flood and drip-irrigated soil.** Box plot showing differential enrichment of microbial taxa with boxes in orange denoting drip-irrigated sample and cyan for flood-irrigated sample in log10 scale. Differences plotted were based on the relative abundance read counts binned to the genera. Besides the 8 shown in this figure, a list of 513 taxa at the genus level were found to be differentially enriched, and their corresponding *p*-value can be found in the supplementary data.

**
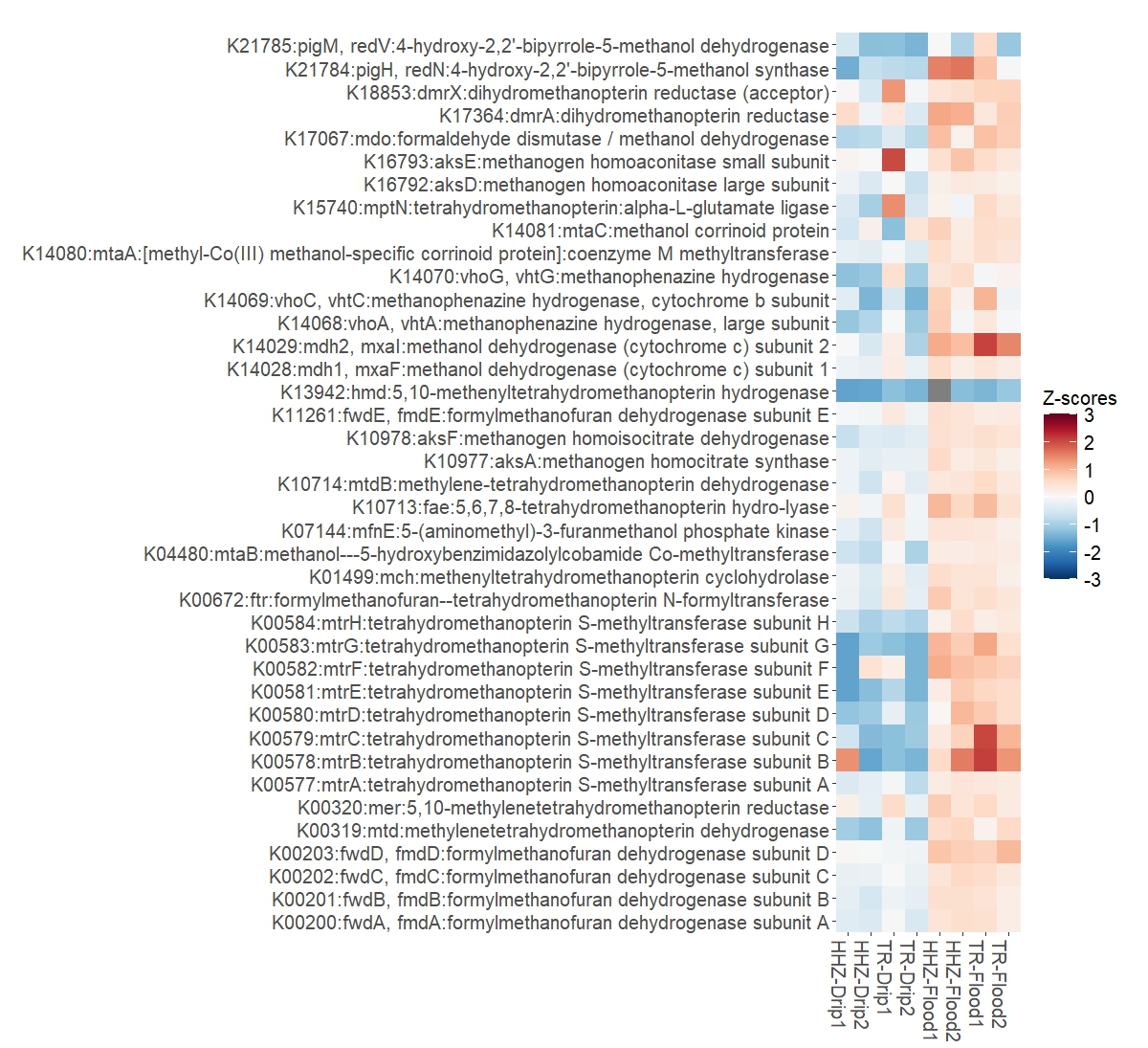
**

**Figure S5. Abundance of key methanogenesis-related genes predicted from soil metagenomes.** The heatmap displays the normalised Z-scores of genes expressed as Reads Per Kilobase per Million mapped reads involved in methane metabolism under continuous flooding versus drip irrigation. Higher Z-scores are depicted in red and lower Z-scores in blue.


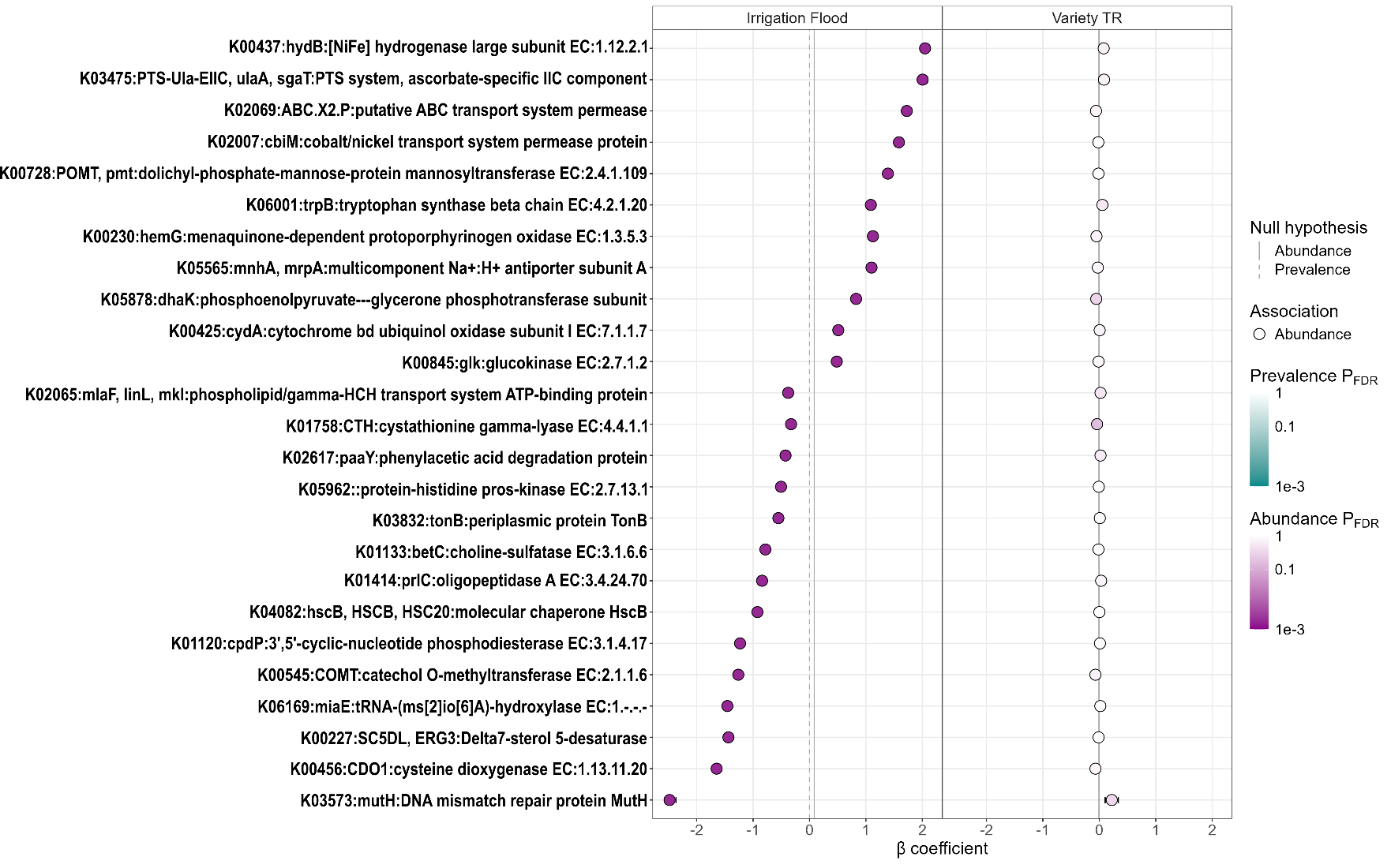


**Figure S6. Association plot of putative KEGG gene functions in MaAsLin2.**Left panel shows statistically significant list of functional genes that are up-regulated and down-regulated in flood irrigation based on beta-coefficient while right panel shows that there are no significant differences in the same set of functional genes between Huanghuazhan and Temasek rice varieties.


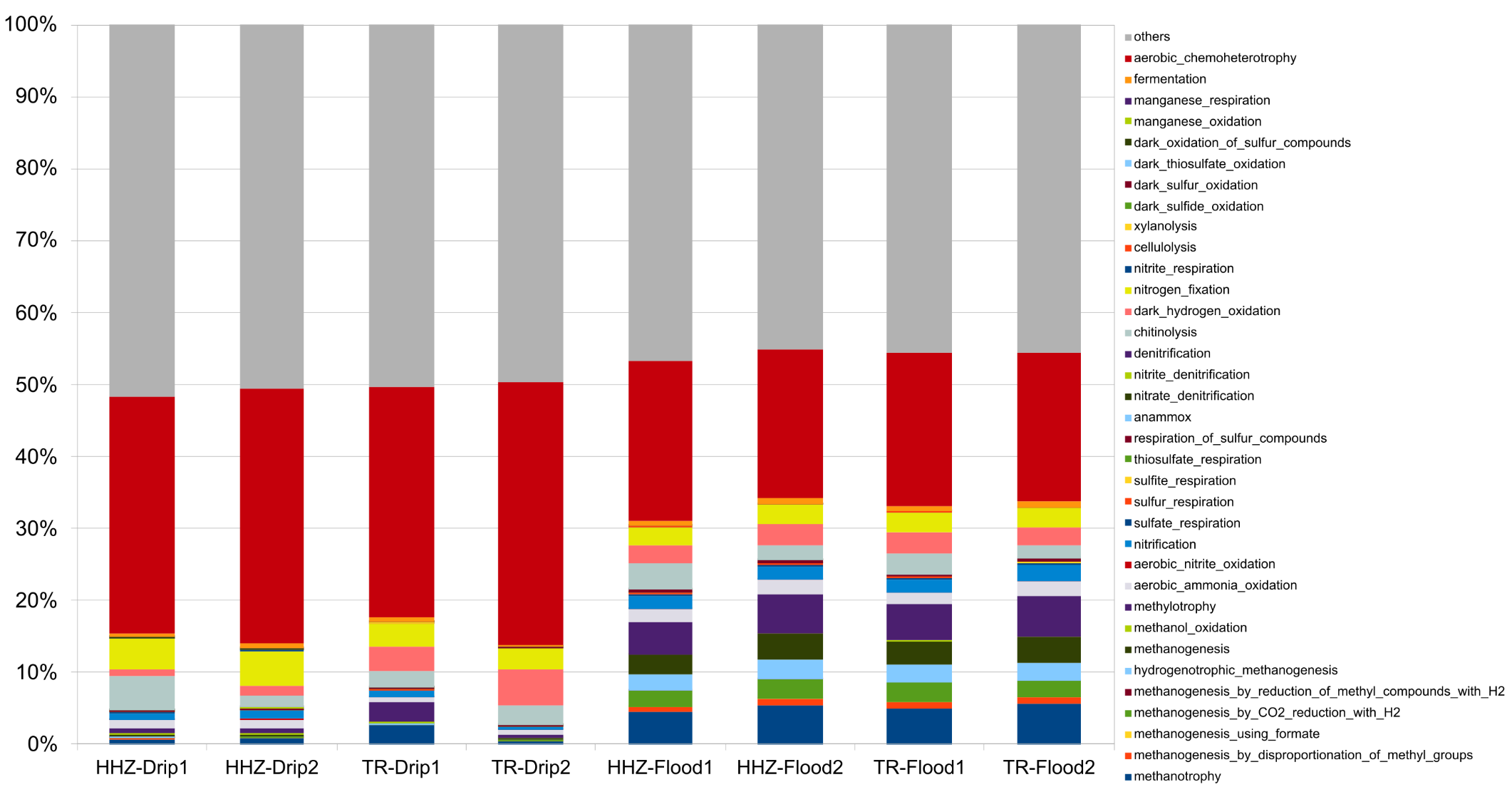


**Figure S7. Relative abundance of key microbial functional categories predicted by FAPROTAX.**The analysis showed predicted proportions of functional categories for both HHZ and TR rice varieties under drip and flood conditions. Stacked bar representation of each functional group is plotted in different colours from bottom to top in sequential order according to the legend.

**
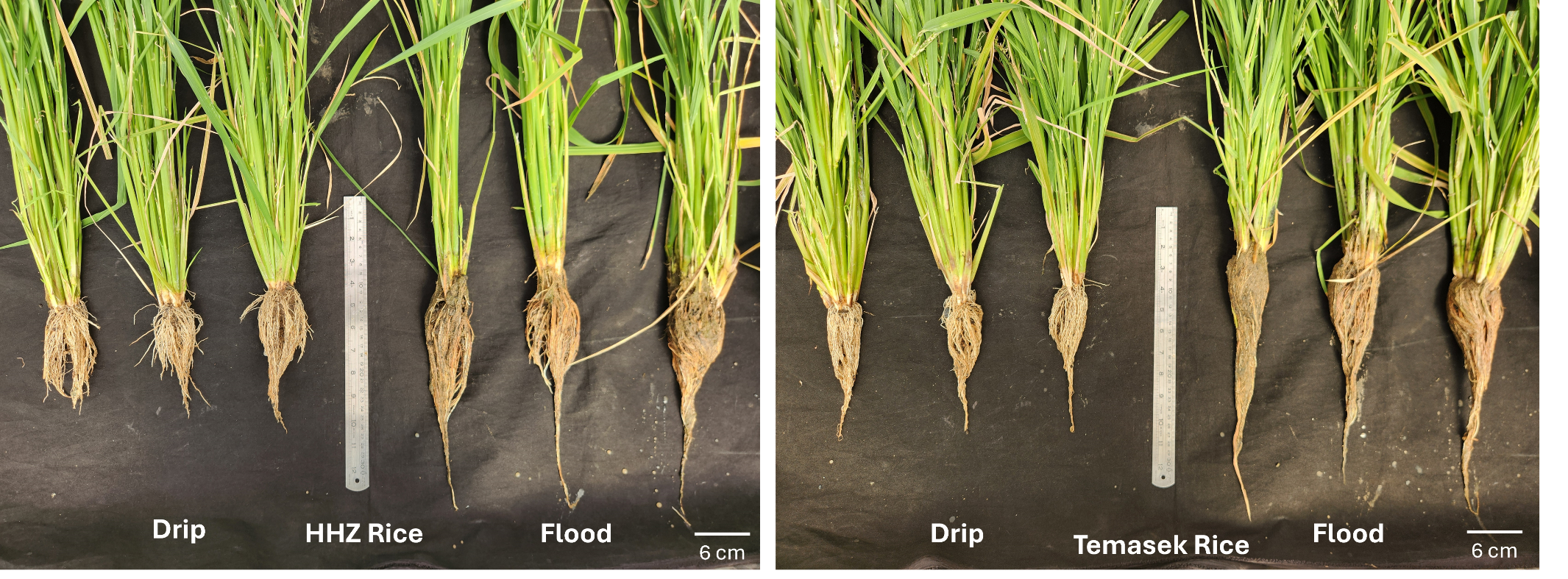
**

**Figure S8. Root length comparison of rice plants under different irrigation regimes.** Rice plants were uprooted at the end of the season. **A.** HHZ and **b.** Temasek rice varieties are displayed in triplicates, with drip-irrigated plants positioned on the left and flood-irrigated plants on the right. 30 cm ruler is placed in the middle as a relative scale bar to determine the root length of the plants. Welch t-test statistical analysis was also performed, and the root length was found to be significantly different between drip and flood irrigation (p < 0.05) as shown in Table S6.

**SUPPLEMENTARY TABLES**

**Table S1. Methane emission is significantly reduced in drip-irrigated rice.** Methane readings obtained were measured in CH₄ m⁻² day⁻¹ over the 11 weeks of rice cultivation. Percentage reduction was calculated as cumulative gathered over the entire course of 11 weeks. Statistical t-test comparisons were performed, and p-value is as shown in the table.

| **Week** | **HHZ_Flood** | **HHZ_Drip** | **TR_Flood** | **TR_Drip** |
| --- | --- | --- | --- | --- |
| 1 | 133.77 | 6.14 | 128.75 | 4.74 |
| 2 | 651.08 | 28.56 | 2362.85 | 8.93 |
| 3 | 725.69 | 130.51 | 1194.63 | 2.60 |
| 4 | 1283.75 | 28.47 | 1237.05 | 56.28 |
| 5 | 749.88 | 20.93 | 1254.54 | 14.70 |
| 6 | 863.65 | 21.77 | 597.22 | 15.91 |
| 7 | 757.04 | 12.00 | 644.39 | 0.00 |
| 8 | 1056.12 | 16.84 | 838.90 | 47.54 |
| 9 | 742.71 | 31.44 | 797.88 | 61.02 |
| 10 | 878.07 | 4.65 | 705.78 | 0.00 |
| 11 | 580.94 | 24.65 | 651.36 | 12.28 |
| Sum | 8422.71 | 325.96 | 10413.36 | 224.01 |
| Percent Reduction | 96% | | 98% | |
| p-value | 6.067e-06 | | 0.0003258 | |

**Table S2 | *mcrA* gene copy number in soil samples collected from the indicated varieties cultivated under drip and flood irrigation.** Duplicate soil samples were collected biweekly and quantitative PCR was performed to determine the Cq value. The corresponding *mcrA* gene copy number were derived from standard curve of y=-2.02 log Cq + 28.814 and normalised to 1 gram of soil.

| **Date** | **Week** | **Variety** | **Irrigation** | **mcrA_Cq1** | **mcrA_Cq2** | **mcrA_CpNo1** | **mcrA_CpNo2** |
| --- | --- | --- | --- | --- | --- | --- | --- |
| 3-Apr-24 | W1 | HHZ | Drip | 29.85 | 30.42 | 338.1356093 | 186.2087 |
| 3-Apr-24 | W1 | HHZ | Flood | 28.43 | 28.61 | 1494.670471 | 1238.019 |
| 3-Apr-24 | W1 | TR | Drip | 27.25 | 26.03 | 5139.360602 | 18427 |
| 3-Apr-24 | W1 | TR | Flood | 27.71 | 26.71 | 3175.544345 | 9044.064 |
| 17-Apr-24 | W3 | HHZ | Drip | 31.46 | 32.89 | 62.70074878 | 14.03695 |
| 17-Apr-24 | W3 | HHZ | Flood | 23.87 | 26.62 | 176714.7201 | 9937.399 |
| 11-Apr-24 | W3 | TR | Drip | 25.23 | 26.18 | 42568.75112 | 15749.72 |
| 11-Apr-24 | W3 | TR | Flood | 20.67 | 20.39 | 5032898.615 | 6746692 |
| 24-Apr-24 | W5 | HHZ | Drip | 22.34 | 23.23 | 876450.257 | 345288.3 |
| 24-Apr-24 | W5 | HHZ | Flood | 19.39 | 19.69 | 19214821.91 | 14036949 |
| 24-Apr-24 | W5 | TR | Drip | 24.57 | 25.22 | 84935.82491 | 43016.63 |
| 24-Apr-24 | W5 | TR | Flood | 19.26 | 19.07 | 22015435.09 | 26859066 |
| 2-May-24 | W7 | HHZ | Drip | 25.13 | 25.24 | 47265.63039 | 42125.54 |
| 2-May-24 | W7 | HHZ | Flood | 20.91 | 21.12 | 3914959.492 | 3142481 |
| 8-May-24 | W7 | TR | Drip | 26.18 | 26.45 | 15749.71601 | 11872.59 |
| 8-May-24 | W7 | TR | Flood | 20.17 | 19.68 | 8493582.491 | 14184636 |
| 29-May-24 | W9 | HHZ | Drip | 22.93 | 23.01 | 472656.3039 | 434692.2 |
| 29-May-24 | W9 | HHZ | Flood | 19.94 | 20.08 | 10805288.42 | 9332543 |
| 29-May-24 | W9 | TR | Drip | 25.07 | 25.5 | 50328.98615 | 32089.55 |
| 29-May-24 | W9 | TR | Flood | 19.86 | 19.9 | 11748975.55 | 11267257 |
| 5-Jun-24 | W11 | HHZ | Drip | 29.91 | 30.59 | 317.5544345 | 155.8573 |
| 5-Jun-24 | W11 | HHZ | Flood | 20.88 | 21.29 | 4039835.083 | 2630268 |
| 5-Jun-24 | W11 | TR | Drip | 25.63 | 25.95 | 28007.3957 | 20036.33 |
| 5-Jun-24 | W11 | TR | Flood | 19 | 19.16 | 28900748.51 | 24444537 |

**Table S3. Assembled metagenome contigs statistics.** Number of predicted ORFs, rRNAs, KEGG, COG, ARG-OAP3 and BACMET annotations for individual samples and assembled contigs.

| **Sample** | **Number of ORFs** | **Number of rRNAs** | **Number of tRNAs/tmRNAs** | **ORFs by Prodigal** | **ORFs by barrnap** | **KEGG annotations** | **COG annotations** | **ARG-OAP3 annotations** | **BACMET annotations** |
| --- | --- | --- | --- | --- | --- | --- | --- | --- | --- |
| Assembly | 12900829 | 16102 | 47814 | 12836913 | 16102 | 5998667 | 8245909 | 180543 | 306991 |
| HHZ-Drip1 | 6623940 | 8891 | 28964 | 6586085 | 8891 | 3450521 | 4609061 | 115801 | 198373 |
| HHZ-Drip2 | 6692195 | 8876 | 29152 | 6654167 | 8876 | 3387647 | 4548356 | 113620 | 195983 |
| HHZ-Flood1 | 7157346 | 10844 | 32017 | 7114485 | 10844 | 3933432 | 5246477 | 131724 | 224493 |
| HHZ-Flood2 | 6790258 | 10470 | 30588 | 6749200 | 10470 | 3741250 | 4991640 | 126300 | 215413 |
| TR-Drip1 | 6594300 | 9088 | 29902 | 6555310 | 9088 | 3549437 | 4727825 | 118784 | 204777 |
| TR-Drip2 | 6385184 | 8398 | 27412 | 6349374 | 8398 | 3256275 | 4365825 | 109634 | 188454 |
| TR-Flood1 | 6470414 | 10306 | 29306 | 6430802 | 10306 | 3620119 | 4805854 | 122840 | 209942 |
| TR-Flood2 | 6217262 | 9862 | 28264 | 6179136 | 9862 | 3440441 | 4581045 | 117160 | 200016 |

**Table S4 | Alpha diversity of soil microbiomes.** Chao1 index indicated that there are higher number of species observed in flooded than drip soil. Additionally, Shannon and Simpson indices suggest that there is no difference in diversity between flooded and drip irrigated soil.

| **Variety** | **Irrigation** | **Replicate** | **Chao1** | **Shannon (H)** | **Simpson (D)** |
| --- | --- | --- | --- | --- | --- |
| HHZ | Drip | 1 | 1938.356 | 3.817144 | 0.941835 |
| HHZ | Drip | 2 | 1939.054 | 3.723193 | 0.935281 |
| HHZ | Flood | 1 | 2289.52 | 4.5047 | 0.973987 |
| HHZ | Flood | 2 | 2253.539 | 4.434425 | 0.972119 |
| TR | Drip | 1 | 1886.874 | 3.617447 | 0.929599 |
| TR | Drip | 2 | 1888.023 | 3.752185 | 0.933789 |
| TR | Flood | 1 | 2217.34 | 4.539406 | 0.975476 |
| TR | Flood | 2 | 2187.991 | 4.401419 | 0.970731 |

**Table S5 | t-test statistics performed on the richness and diversity indices.** Welch t-test were performed on Chao1, Shannon and Simpson indices for a. drip irrigated versus flooded and b. HHZ versus Temasek rice.

a.

| **Group** | **Mean** | **t-statistic** | **df** | **p-value** |
| --- | --- | --- | --- | --- |
| Drip (Chao1) | 1913.077 | -12.212 | 5.2504 | 4.698e-05*** |
| Flood (Chao1) | 2237.097 |  |  |  |
| Drip (Shannon) | 3.727492 | -14.209 | 5.5976 | 1.323e-05*** |
| Flood (Shannon) | 4.469988 |  |  |  |
| Drip (Simpson) | 0.9351260 | -13.829 | 3.9808 | 0.0001633*** |
| Flood (Simpson) | 0.9730783 |  |  |  |

b.

| **Species** | **Mean** | **t-statistic** | **df** | **p-value** |
| --- | --- | --- | --- | --- |
| HHZ (Chao1) | 2105.117 | 0.45271 | 5.9819 | 0.6667 |
| Temasek Rice (Chao1) | 2045.057 |  |  |  |
| HHZ (Shannon) | 4.119865 | 0.13758 | 5.9099 | 0.8951 |
| Temasek Rice (Shannon) | 4.077614 |  |  |  |
| HHZ (Simpson) | 0.9558055 | 0.21736 | 5.8178 | 0.8354 |
| Temasek Rice (Simpson) | 0.9523987 |  |  |  |

**Table S6 | Root length measurements**. The length of plant roots was measured using the line drawing tool in ImageJ. Triplicates were collected for a. HHZ and b. Temasek rice.

| **Variety** | **Replicate** | **Drip (cm)** | **Flood (cm)** |
| --- | --- | --- | --- |
| HHZ | 1 | 12.0 | 23.9 |
| HHZ | 2 | 12.7 | 25.0 |
| HHZ | 3 | 16.4 | 26.2 |
| Average | | 13.7 | 25.0 |
| p-value (significant) p < 0.05 | | | 0.005607*** |

b.

| **Variety** | **Replicate** | **Drip (cm)** | **Flood (cm)** |
| --- | --- | --- | --- |
| Temasek | 1 | 16.0 | 31.5 |
| Temasek | 2 | 15.1 | 25.6 |
| Temasek | 3 | 16.6 | 30.4 |
| Average | | 15.9 | 29.2 |
| p-value (significant) p < 0.05 | | | 0.01417** |
